# Accurate Relativistic Real-Time Time-Dependent Density Functional Theory for Valence and Core Attosecond Transient Absorption Spectroscopy

**DOI:** 10.1021/acs.jpclett.2c03599

**Published:** 2023-02-09

**Authors:** Torsha Moitra, Lukas Konecny, Marius Kadek, Angel Rubio, Michal Repisky

**Affiliations:** † Hylleraas Centre for Quantum Molecular Sciences, Department of Chemistry, UiT The Arctic University of Norway, 9037 Tromsø, Norway; ‡ Max Planck Institute for the Structure and Dynamics of Matter, Center for Free Electron Laser Science, Luruper Chaussee 149, 22761 Hamburg, Germany; § Department of Physics, Northeastern University, Boston, Massachusetts 02115, United States; ∥ Algorithmiq Ltd., Kanavakatu 3C, FI-00160 Helsinki, Finland; ⊥ Center for Computational Quantum Physics (CCQ), The Flatiron Institute, 162 Fifth Avenue, New York New York 10010, United States; ∇ Nano-Bio Spectroscopy Group, Departamento de Física de Materiales, Universidad del País Vasco, 20018 San Sebastian, Spain; # Department of Physical and Theoretical Chemistry, Faculty of Natural Sciences, Comenius University, 84104 Bratislava, Slovakia

## Abstract

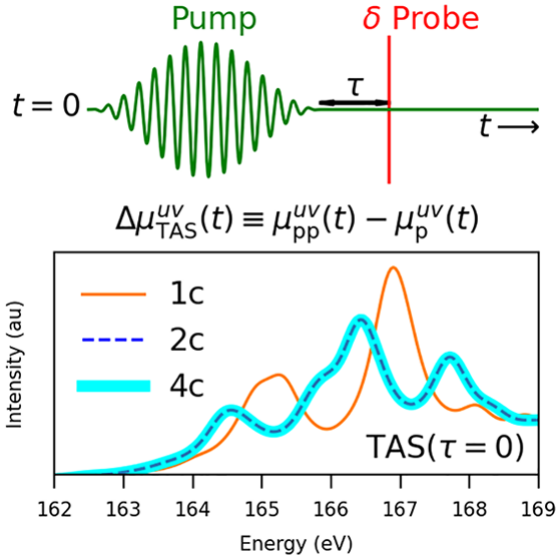

First principles theoretical modeling of out-of-equilibrium processes observed in attosecond pump–probe transient absorption spectroscopy (TAS) triggering pure electron dynamics remains a challenging task, especially for heavy elements and/or core excitations containing fingerprints of scalar and spin–orbit relativistic effects. To address this, we formulate a methodology for simulating TAS within the relativistic real-time, time-dependent density functional theory (RT-TDDFT) framework, for both the valence and core energy regimes. Especially for TAS, full four-component (4c) RT simulations are feasible but computationally demanding. Therefore, in addition to the 4c approach, we also introduce the atomic mean-field exact two-component (amfX2C) Hamiltonian accounting for one- and two-electron picture-change corrections within RT-TDDFT. amfX2C preserves the accuracy of the parent 4c method at a fraction of its computational cost. Finally, we apply the methodology to study valence and near-L_2,3_-edge TAS processes of experimentally relevant systems and provide additional physical insights using relativistic nonequilibrium response theory.

Transient absorption spectroscopy (TAS) is a powerful nonlinear technique for investigating ultrafast state-resolved processes and electronic superposition using two pulses, viz., pump and time-delayed probe pulse.^1−3^ TAS offers additional degrees of freedom, namely, pump field features such as shape, amplitude, carrier frequency, direction, and pump–probe time delays, which are absent in conventional spectroscopy. The effect of the time delay between the pulses is most commonly followed experimentally.^4,5^ The emphasis has long been on the femto- to picosecond time scales, which involved time-resolving processes initiated by nuclear motions.^6−9^ However, with the advent of attosecond pulses, there has been ever-increasing interest in studying subfemtosecond time scale processes, focusing on the motion of electrons on their natural time scale, with a minimal influence of nuclear degrees of freedom.^2,10−19^ Simultaneously, the generation of intense isolated soft X-ray free electron laser pulses with subfemtosecond temporal widths has been achieved recently, promoting investigations of attosecond-resolved spectroscopies involving core orbitals.^20,21^ Due to their characteristic element specificity and unprecedented temporal resolution, ultrafast X-ray spectroscopies are emerging as indispensable tools for biological and material sciences.^22,23^ In particular, X-ray transient absorption spectroscopy (XTAS), which employs short X-ray probe pulses, is now extensively being used to investigate the electron dynamics of molecules and solid-state systems.^7,22−27^ This dictates the need to develop reliable theoretical tools to aid the interpretation and prediction of such phenomena.

With regard to simulating the response of the laser pulses relevant to pump–probe spectroscopies, the real-time (RT) formalism offers a straightforward approach^28−46^ over response-theory-based methods as the former is applicable for a large range of field intensities and resembles the experimental setup in a natural way. Addressing core-level spectroscopies has added complexities, as it requires the inclusion of scalar (SC) and spin–orbit (SO) relativistic effects, which are most reliably described by multicomponent relativistic quantum chemical methods. Here, the “gold standard” is the four-component (4c) methodology including both SC and SO effects variationally via the one-electron Dirac Hamiltonian in combination with instantaneous Coulomb interactions among the particles. Since the 4c treatment can be time-consuming, especially for RT simulations, researchers have focused on the development of approximate 2c Hamiltonians.^47^ An approach that has gained wide popularity in recent years is the exact two-component (X2C) Hamiltonian as it reduces the original 4c problem by half, requiring only a few simple algebraic manipulations.^48−53^ There exist several variants of the X2C Hamiltonian, ranging from a pure one-electron X2C (1eX2C) Hamiltonian where two-electron (2e) interactions are entirely omitted from the X2C decoupling transformation^47,54^ to a molecular mean-field X2C (mmfX2C) Hamiltonian where the decoupling is performed *after* 4c molecular self-consistent field (SCF) calculations.^55^ In between, there are several X2C Hamiltonian models that extend 1eX2C by including 2e interactions approximately via (i) element- and angular-momentum-specific screening factors in the evaluation of one-electron SO integrals;^56,57^ (ii) a mean-field SO approach^58^ which has been the basis for the widely popular AMFI module;^59^ and (iii) an approach that exploits atomic model densities obtained within the framework of Kohn–Sham DFT.^60,61^ The screening factors of type (i) are sometimes referred to as “Boettger factors” or as the (modified) screened nuclear spin–orbit approach ((m)SNSO).^62,63^ Recently, an atomic mean-field (amfX2C) and an extended atomic mean-field (eamfX2C) approach have been presented within the X2C framework,^64^ extending some of earlier ideas of Liu and Cheng^65^ by comprising the full 2e SO and SC corrections, regardless whether they arise from the Coulomb, Coulomb–Gaunt, or Coulomb–Breit Hamiltonian. Moreover, this ansatz takes into account the characteristics of the underlying correlation framework, viz., wave function theory or (KS-)DFT, which enables tailor-made exchange–correlation (xc) corrections to be introduced.^64^ While all above-mentioned relativistic methods lie within the static time-independent regime, extensions to the RT framework^66^ were recently formulated both at the 4c^67−69^ and 2c^54,70,71^ levels.

In this letter, we present a novel theoretical methodology to address several fascinating characteristics associated with TAS and its simulation. First, to understand the physics governing TAS, we generalize the nonequilibrium response theory formalism to incorporate complex orbitals necessary for relativistic theory. This allows us to interpret unique spectral observations inherent to TAS. Next, to provide a first-principles computational approach for the simulation of TAS across the entire periodic table and/or core atomic region(s), we implement a relativistic variant of RT time-dependent density functional theory (TDDFT) based on 4c Dirac–Coulomb Hamiltonian. This allows us to account for SC and SO relativistic effects variationally, thereby significantly broadening the applicability of the tool. Since the gold standard 4c method is still computationally demanding and popular one-electron 1eX2C is not sufficiently accurate in comparison to its 4c reference,^72^ finally we formulate and implement for the first time a simple yet numerically accurate amfX2C Hamiltonian in the context of the RT framework. It incorporates all spin-free and spin-dependent relativistic picture-change corrections originating from X2C transformation, giving remarkable agreement with reference 4c results. Consequently, we apply the amfX2C Hamiltonian to provide physical insights into the TAS near valence and L_2,3_ edge for experimentally relevant systems.

In order to lay the theoretical foundations for describing pump–probe spectroscopy, we consider in this work the nonoverlapping regime in which the probe field/pulse  is applied at or after the end of the pump pulse . The pump pulse takes the form
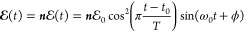
1and is characterized by the amplitude , shape (cos^2^-enveloped sin function), carrier frequency ω_0_, time duration *T*, carrier–envelope phase (CEP) ϕ, and polarization along the unit vector ***n***. The pump pulse is centered at *t*_0_ and is zero (inactive) outside of the window of size *T*, i.e., for  and . In practice, the duration of the pump is chosen to be an integer number of carrier periods, the time *t* = 0 corresponds to the onset of the pump, i.e., , and the CEP is set to 0. For the probe pulse, we use the analytical form of a delta field^67^
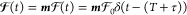
2characterized by its amplitude and polarization direction ***m*** and positioned at *T* + τ, where τ is a time delay between the pump and probe pulses.

To gain physical insight into pump–probe processes, it is instructive to examine them from the point of nonequilibrium response theory.^73^ First, we derive the nonperturbative form of the response to the probe field applied to the nonstationary (out-of-equilibrium) state  generated by the previous pump pulse lasting from time 0 to *T*. The time evolution of a system after the end of the pump is described by the Hamiltonian
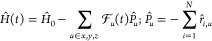
3where *Ĥ*_0_ is the static Hamiltonian describing the *N* electron system itself and  denotes the coupling of the molecular system to the probe pulse via the electric dipole operator ***P̂***. Note that we have assumed the dipole approximation, and we work in the length gauge in this paper. The wave function |Ψ(*t*)⟩ for times *t* > T + τ can be expressed using unitary evolution operators^74^ that propagate the state  from time *t* = *T*. To isolate the singularity caused by the δ-type probe pulse, we split the time propagation into three parts using a sequence of evolution operators. First, we propagate from the time *t* = *T* to *t* = *T* + τ. Here, the evolution operator has the simple form of e^–i*Ĥ*_0_τ^ (where *T* cancels out), since both the pump and probe pulses are not active in this interval, and the time propagation is determined only by the static Hamiltonian *Ĥ*_0_. Second, we handle the probe perturbation by propagating the wave function on the infinitesimal time interval from *t* = *T* + τ – ϵ to *t* = *T* + τ + ϵ as ϵ → 0. Such a “step” propagation can be expressed in the closed form^67^ as e^–i*Q̂*_*v*_^, where . Third, the evolution from *t* = *T* + τ to an arbitrary *t* is, as in the first case, determined by the static Hamiltonian as e^–*iĤ*_0_(*t*–*T*–τ)^. Hence, the wave function is given by
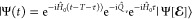
4On the basis of stationary eigenstates |Φ_*j*_⟩ of the static Hamiltonian *Ĥ*_0_, the state  is expressed as a linear combination
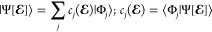
5Using the resolution-of-the-identity ∑_*j*_|Φ_*j*_⟩ ⟨Φ_*j*_| = 1̂, the nonperturbative electric dipole response to the probe pulse  can be written in the frequency domain as
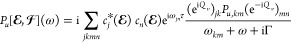
6where ω_*jk*_ ≡ ϵ_*j*_ – ϵ_*k*_ is the difference between the energies of the stationary states; for an operator *Â*, *A*_*jk*_ ≡ ⟨Φ_*j*_|*Â*|Φ_*k*_⟩ is its matrix element; *P*_*u*_(ω) = ∫_*T* + τ_^∞^*P*_*u*_(*t*)e^(i*ω*–Γ)(*t*–*T*–τ)^ d*t*, and a damping parameter Γ is introduced to regularize the Fourier integral over oscillating functions and facilitate its practical evaluation in simulations with a finite time length. Using the expansion  in eq 6 allows us to understand the most dominant effects on the resulting spectra, i.e., those that appear even in the weak probe field limit. After rearranging the summation indices, we obtain the first terms
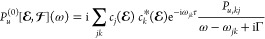
7and
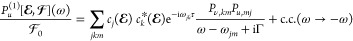
8where c.c.(ω → −ω) labels the complex conjugation while flipping the sign of the frequency. We note that in the relativistic theory presented here, the stationary wave functions are complex in general. As a consequence, the matrix elements *P*_*ν*,*km*_ can no longer be assumed to be real. Expressions in eqs 7 and 8 contrast with the equilibrium response theory where a response function is defined in terms of a specific eigenstate of *Ĥ*_0_, e.g.,  and , assuming this would yield the equilibrium response functions.

The process of electronic absorption, which is of interest here, is associated with the imaginary part of the electric dipole–electric dipole response function in the frequency domain ().^75^ The resulting expression for the response function was presented by other authors,^76^ while assuming that the eigenstates |Φ_*j*_⟩ and hence the matrix elements *P*_*v*,*km*_ are real-valued. However, this is not the case in the relativistic theory, and we here proceed by deriving the relativistic extension for the response function without making this assumption. Let ϕ_*j*_ denote the phase of the complex amplitudes *c*_*j*_, i.e., *c*_*j*_ = |*c*_*j*_|e^i*ϕ*_*j*_^. By defining Θ_*jk*_(τ) ≡ ϕ_*j*_ – ϕ_*k*_ – ω_*jk*_τ and using , where  and  represent Lorentzian and Rayleigh line shape functions, respectively, we can rearrange the terms in eq 8 and extract the imaginary part as
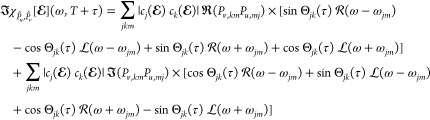
9The first part of this expression proportional to the real part  coincides with the result of Walkenhorst et al.^76^ for . The second part is nonzero only for theories that lead to complex stationary states, e.g., when the Hamiltonian *Ĥ*_0_ is not real-valued, such as the relativistic theory with SO effects included. We note here that this distinction between the theories based on complex and real orbitals is a unique feature of the nonequilibrium response function, and the differences between the formulations vanish when the nonstationary state  is replaced with a single eigenstate (for spatially isotropic values ). Finally, we mention that the diagonal terms *j* = *k* in the sums in eq 9 can be isolated to study separately the time-delay-independent contribution and interference term that depend on the time delay τ. The interference term is a signature of the nonstationary state, leading to the spectral dependence on *τ*. Θ_*jk*_ combines the real and imaginary parts of the response function and interchanges the  with  line shapes and vice versa. The imprints of eq 9 can be directly connected to the simulated spectral observations and are discussed later.^73,76^

Instead of using the nonequilibrium response theory to obtain TAS spectra, we work directly in the time domain and evolve a molecular system of interest by the Liouville–von Neumann equation of motion (EOM). For Kohn–Sham TDDFT in an orthonormal basis, the EOM takes the form
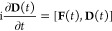
10where **D**(*t*) is the time-dependent reduced one-electron density matrix describing the state of the system at time *t* and **F**(*t*) is the Fock matrix driving the time evolution and characterizing the molecular system itself as well as its interaction with the external pump–probe fields. In practice, eq 10 is solved numerically by propagating **D**(*t*) in time as detailed in refs (31), (67), (77), and (78) as well as in section S1.

The theoretical modeling of core-level spectroscopies is a challenging task because the spectra feature a fine structure due to scalar (SC) and spin–orbit (SO) relativistic effects.^68,79−83^ With this in mind, we have extended our recent probe-only four-component (4c) RT-TDDFT implementation^67,68,84^ to the realm of pump–probe experiments. Assuming an orthonormalized atomic orbital (AO) basis, the 4c Fock operator suitable for TAS has the following matrix form
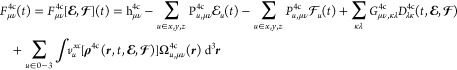
11where terms on the right-hand side include the one-electron Dirac operator (**h**^4c^), particle–field interactions via the electric dipole moment operator (**P**_*u*_^4c^), two-electron (2e) Coulomb and exchange interactions, and the exchange–correlation (xc) contribution. Here, the 2e term involves the matrix of generalized antisymmetrized electron repulsion integrals
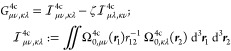
12with a scalar scaling factor ζ for exchange interaction, whereas the xc term requires a noncollinear xc potential *v*_*u*_^*xc*^, given within a generalized gradient approximation (GGA) by derivatives of the nonrelativistic xc energy density (ε^*xc*^) with respect to the 4c electronic charge density (ρ_0_^4c^) and spin densities (ρ_1–3_^4c^), and their gradients:
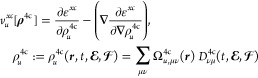
13More details about our noncollinear extension of nonrelativistic xc potentials are available in ref (85). In eqs 12 and 13, **Ω**_*u*_^4c^ stands for the matrix of overlap distribution functions^84^
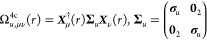
14defined as the product of orthonormal 4c AO basis functions ***X***_μ_(***r***) := ***X***_μ_^RKB^(***r***) fulfilling a restricted kinetic balance (RKB) condition in their small component^86^ and 4c operators associated with the electronic charge density (**Σ**_0_) and spin densities (**Σ**_1–3_). The latter ones are defined via the 2 × 2 zero matrix (**0**_2_), identity matrix (**σ**_0_), and Pauli spin matrices (**σ**_1_, **σ**_2_, **σ**_3_). Note that prior to their use in eq 10, both Fock and the density matrix are transformed on the basis of ground-state molecular orbitals, obtained from the solution of self-consistent field equations.

While the simulation of XAS by means of the full 4c RT-TDDFT method is nowadays feasible but computationally challenging,^68^ its further extension toward pump–probe experiments poses an additional computational burden. Therefore, there is interest in developing approximate relativistic methods enabling RT calculations in the two-component (2c) regime while maintaining the accuracy of the parent 4c approach. Hence, alongside the 4c method that serves as a gold-standard reference, we put forth a first formulation and implementation of an atomic mean-field exact two-component Hamiltonian (amfX2C) within the realm of RT-TDDFT and apply it to simulate (X)TAS spectra. As proven for self-consistent field calculations, the simple yet numerically accurate amfX2C approach accounts for so-called SC and SO two-electron (2e) and exchange–correlation (xc) picture-change (PC) effects that arise from the X2C transformation, in contrast to the commonly used one-electron X2C (1eX2C) Hamiltonian.^64^ Furthermore, as demonstrated on simple XAS spectra of transition-metal and actinide compounds, the absence of these PC effects in 1eX2C results in a substantial overestimation of L- and M-edge SO splittings, whereas the amfX2C Hamiltonian reproduces all essential spectral features such as shape, position, and SO splitting in excellent agreement with 4c references while offering more than a 7-fold speed-up.^72^ A similar acceleration was reported previously on RT-TDDFT based on 1eX2C.^54^

The central idea of our RT-TDDFT amfX2C approach is the matrix transformation of the original 4c EOM in eq 10 to its diagonally dominant form using a static (time-independent) unitary matrix **U**. By maintaining only the large-component–large-component (LL) block of the transformed 4c EOM, one arrives at the 2c EOM^54,72^
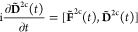
15with the so-called 2c PC transformed Fock and density matrix
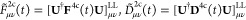
16Consistent with our previous works, we use notation with tildes to indicate picture-change transformed quantities.^64,72^ Of significant importance is the observation that the *correctly* transformed 2c Fock matrix involves not only the PC transformed density matrix (**D̃**^2c^) but also the overlap distribution matrix as well as one- and two-electron integrals.^64,72^ In connection to eq 11, our 2c RT-TDDFT (X)TAS reads
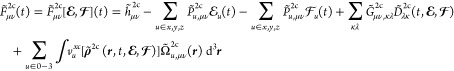
17Note, however, that the presence of the picture-change transformed overlap distribution matrix (**Ω̃**^2c^) in both 2e and xc interaction terms makes the evaluation of **F̃**^2c^ computationally more demanding than the original 4c Fock matrix. Therefore, in line with the amfX2C approach introduced originally for the static (time-independent) SCF case^64^ and extended later to the response theory formalism involving electric field(s),^72^**F̃**^2c^ in eq 17 can be approximated by a computationally efficient form built with untransformed (without the tilde) overlap distributions (**Ω**^2c^), i.e.,
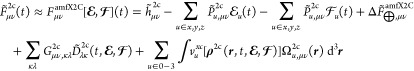
18In fact, eq 18 defines our 2c amfX2C Fock matrix applied in actual RT (X)TAS simulations. Here, the picture-change corrections, defined as the difference between transformed and untransformed 2e and xc interaction terms, are taken into account approximately via Δ**F̃**_⊕_^amfX2C^ and given by a superposition of corresponding static atomic quantities^64,72^
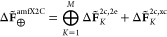
19where
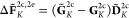
20
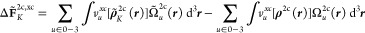
21Here, *K* runs over all atoms in an *M*-atomic system and labels atomic quantities obtained from independent atomic SCF calculations, each performed in the orthonormal AO basis of the *K*th atom. While a theoretical justification enabling us to build Δ**F̃**_⊕_^amfX2C^ from static (time-independent) quantities is available in ref (72), a pseudocode highlighting the essential steps for evaluating Δ**F̃**_⊕_^amfX2C^ is presented in ref (64).

The final TAS spectra are obtained within the RT-TDDFT framework from the differential induced dipole moment
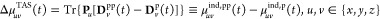
22where μ_*uv*_^ind^(*t*) denotes the expectation value of the dipole operator. The computation of TAS spectra involves performing two simulations for recording the dipole moment at each time step; these simulations and their quantities are denoted by p and pp subscripts, indicating that the real-time propagation used the pump-only pulse and pump together with the probe pulse, respectively. This difference is calculated to isolate the effect of the probe pulse on the nonstationary electronic wavepacket, i.e., to eliminate pump-only dependent terms in eq 7. This procedure is in contrast to the simulations initiated from an equilibrium stationary state (i.e., without the pump), for which eq 7 simplifies to the static dipole moment that is subtracted in eq 22, and the absorption spectra thus require only one time propagation simulation. The differential dipole moment is subsequently transformed to the frequency domain (as in eq 6). Finally, the TAS spectral function *S*^TAS^(ω) is evaluated analogously to its ground-state absorption counterpart as
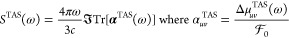
23In eq 23, *c* is the speed of light and Tr is a trace over Cartesian components of the polarizability tensor **α**.

In simulated X-ray spectra, signals originating from transitions between valence and high-lying virtual orbitals can appear. These are nonphysical in calculations using finite atom-centered basis sets, since above-ionization states are ill-described in such cases. To eliminate these spurious peaks, we restrict the probe operator to act only on a selected subset of core and virtual orbitals by zeroing all other elements of the dipole matrix when applying the probe pulse, thus making the nonphysical transitions artificially dipole-forbidden. This technique, called selective perturbation, was introduced in our previous works in the context of XAS calculations using both RT-TDDFT^68^ and damped linear response TDDFT.^83^ Note that no restriction is applied to the pump pulse as that is purposefully tuned to an excitation from valence to low-lying orbitals.

In line with the spectral analysis technique for RT-TDDFT simulations presented in ref (67), we assign the nature of electronic excitations underlying a particular TAS resonance feature by a dipole-weighted transition matrix analysis (DWTA). For a resonant frequency ω′, the Fourier component  of the time-domain signal  contains all information.
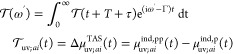
24Here, *a*,*i* runs over occupied and virtual spinors, respectively, and we accounted for the fact that only the time signal after the probe pulse is used for the Fourier transform. We use the differential induced dipole moment (eq 22) at a specific resonance frequency (ω′) to obtain the contribution of individual occupied virtual pairs toward the spectral peak. The  matrix is obtained by averaging over the Cartesian components.

The above-formulated RT-TDDFT methodology was implemented in the RESPECT program^84^ and used to investigate the TAS spectra of two prototypical molecules, ethylene and thiophene, in the valence and core (L_2,3_) energy regions, respectively, employing 1c nonrelativistic, 2c amfX2C, and the 4c Dirac–Coulomb Hamiltonian. Geometries of the systems are given in the SI. The uncontracted aug-cc-pVXZ basis set (X = T(C), D(H) for ethylene and T(S), D(C,H) for thiophene)^87−90^ with the PBE0-40HF hybrid xc functional^91^ including 40% exact exchange contributions is used.^83^ For all nuclei, a finite-sized Gaussian model was used. For core absorption processes, we use the selective perturbation scheme described above, whereby only electric dipole moment contributions originating from the core S 2p_1/2_ and 2p_3/2_ orbitals are considered to be dipole-allowed.^68^ The details of the TAS computational setup are elucidated in Table 1 and Figure S1. Note that the choice of direction of polarization of the pump pulse (***n***) is important as the spectra are sensitive to geometrical features.

**Table 1:** Computational Setup^a^

						RT simulation	
molecule	ω_0_ (au)	*T* (au)	***n***	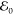 (au)	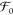 (au)	*n*_step_	*Δt*_step_ (au)
C_2_H_4_	0.2762	341.10	*x*	0.01	0.01	20 000	0.15
C_4_H_4_S	0.2135	441.20	*y*	0.05	0.01	25 000	0.20

a The pump and probe pulse parameters correspond to eqs 1 and 2. The carrier frequency (ω_0_) is tuned to the first bright excited state along the direction of the dipole-allowed transition (***n***). The time duration (*T*) of the pump pulse corresponds to 15 carrier periods. The pump pulse amplitude () is chosen to sufficiently depopulate the ground state. A weak δ probe of amplitude  along all of the Cartesian components is applied. The probed system is evolved for *n*_step_ with *Δt*_step_ length as detailed in section S1, using the convergence criterion for microiterations |**D**^(*n*)^(*t* + *Δt*) – **D**^(*n*+1)^(*t* + *Δt*)| < 10^–6^. These parameters are used unless otherwise stated.

For ethylene, we study the effect of the degrees of freedom specific to pump–probe processes, focusing on the pump pulse strength and pump–probe time delay. First, the effect of pump pulse strength on the TAS spectra is shown in Figure 1. The electronic wavepacket  generated by the pump pulse comprises an admixture of ground and excited electronic states (eq 5), with the effective depopulation of the ground state being proportional to . The ground-state occupancy at time *t* is estimated to be Tr[**D**(0) **D**(*t*) ].^76^ It is evident from Figure 1 that considerable depopulation of the ground state is necessary to differentiate TAS spectra from ordinary ground-state absorption spectra. The negative intensity signal at about 0.3 au and  au in Figure 1a is a hallmark of the nonstationary state involved in this pump–probe process and is a consequence of mixing Lorentzian and Rayleigh line-shape functions as derived in eq 9. TAS spectra of ethylene obtained with  au are in good agreement with the pioneering work by Giovannini et al.^34^ using Octopus code.^92−94^ However, due to different computational setups and lack of experimental references we do not focus on a detailed assignment of the TAS spectral features. For exact theory, the ground-state population at *t* > *T* + τ depends only on τ and pulse intensities and is constant with respect to the simulation time *t*. This can be derived from eqs 4 and 5 as
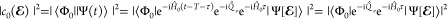
25However, this behavior does not hold for approximate mean-field methods such as HF or DFT, where the exponential term e^–i*Ĥ*_0_(*t*–*T*–τ)^ does not drop out of the integration as a consequence of the implicit time dependence incorporated into the Fock matrix. The resulting oscillations in the ground-state population, as seen in Figure 1b, are artifacts attributed to the use of an adiabatic approximation.^95−97^

**Figure 1:**
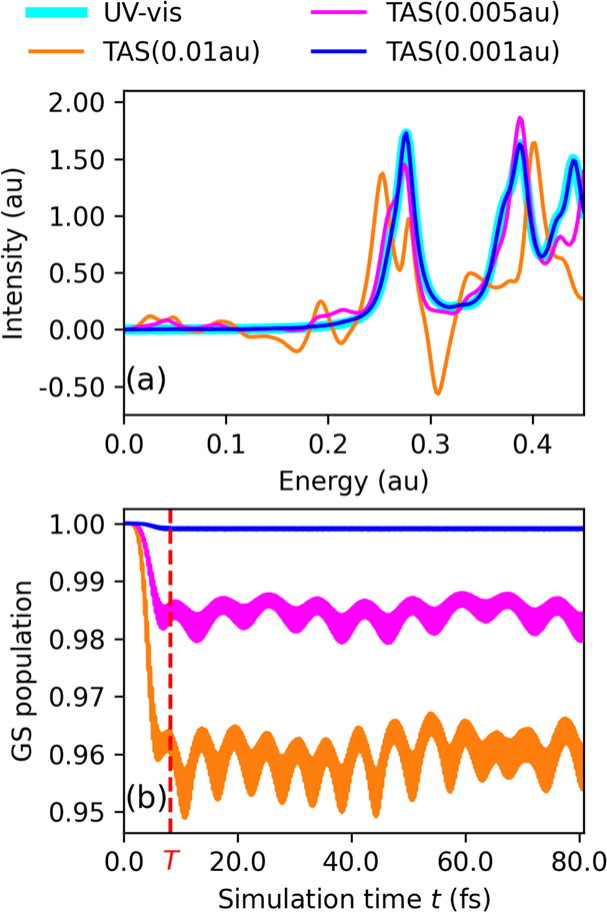
Ethylene. (a) Comparison of UV–vis and TAS spectra with varying , given in parentheses, obtained with the amfX2C Hamiltonian. TAS is computed at τ = 0.0 with a damping parameter Γ = 0.01 au. (b) Variation in the ground-state population as a function of simulation time *t* obtained as Tr[**D**(0) **D**(*t*) ], with pump amplitudes as color coded in (a). *T* (in red) marks the duration of the pump pulse.

From an experimental point of view, the most important degree of freedom in TAS is the pump–probe time delay τ. The influence of τ on TAS for ethylene is depicted in Figure 2c. As the pump pulse is tuned to the first bright state in the ground-state absorption spectrum (**A**′ in Figure 2b), this ground-state peak is split into peaks **A**, **B**, and **C** in the TAS. DWTA analysis of **A**′, **A**, **B**, and **C** (shown in Figure 2d and Figures S3 and S4) shares same dominantly contributing transition of π → π* character involving degenerate spinor pair (15, 16) → (19, 20). Secondary contributions come from transitions involving degenerate spinor pair (13, 14) → (46, 45). Interestingly, peak **D** has completely different character from any of the features obtained in the ground-state absorption spectra, further emphasizing the novelty of this spectroscopic technique to probe states (and therein molecular orbitals) not directly accessible by purely ground- or excited-state absorption.

**Figure 2:**
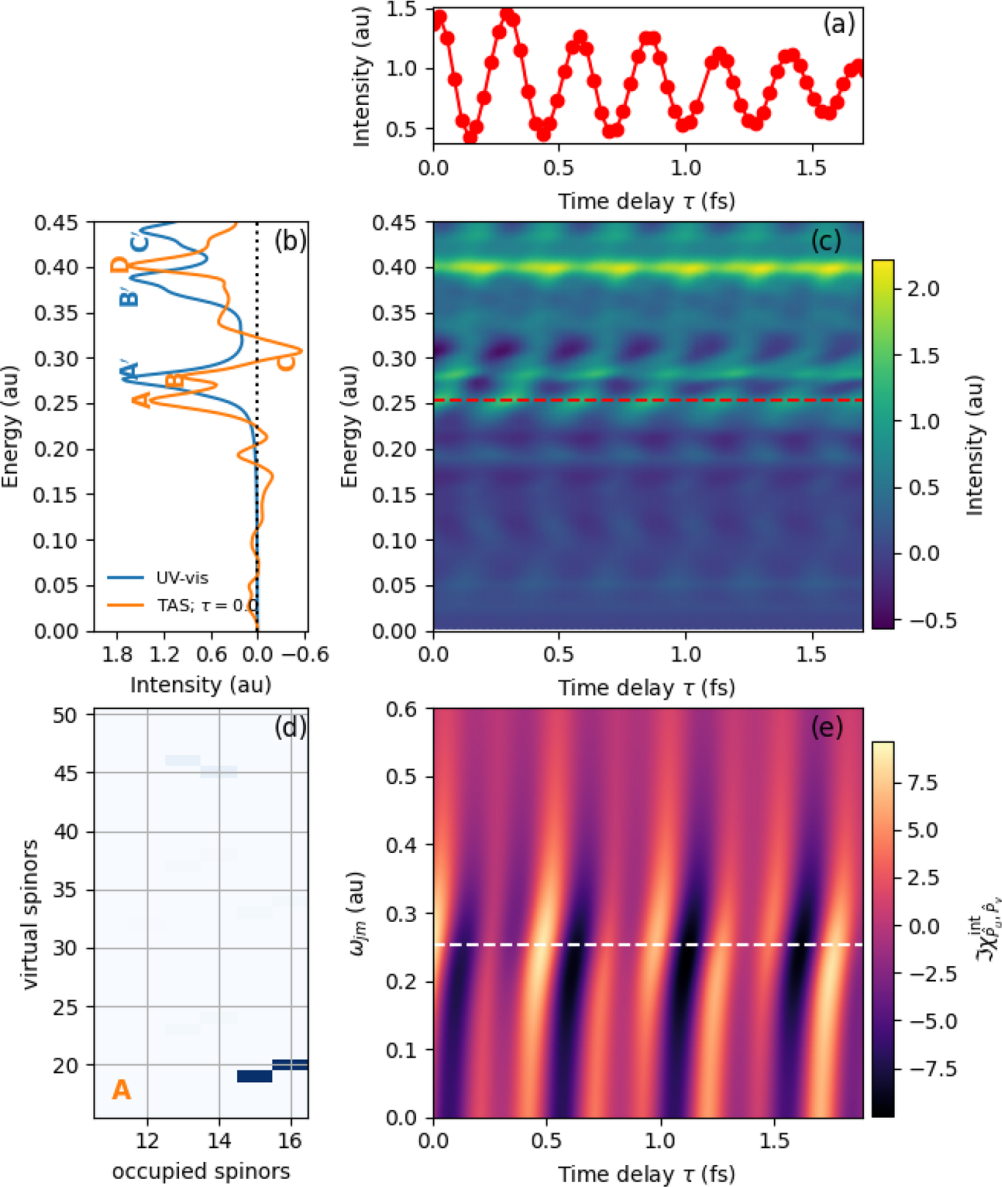
Ethylene. (a) Variation in intensity with τ for the TAS spectral peak at ω_0_ = 0.2534 au. (b) Ground-state absorption and TAS spectra at τ = 0.0. (c) TAS spectra with varying τ. (d) DWTA of TAS peak **A** at ω_0_ = 0.2534 au and τ = 0.0. The intensity of the blue color corresponds to the intensity of the particular excitation. (e)  is given in eq 26 for a degenerate *k*, *j* spinor pair selected as (15, 16) → (19, 20) and (13, 14) → (46, 45) with orbital energy differences of 0.3305 and 0.6039 au, respectively. The white dashed lines correspond to the resonant ω. The 2c amfX2C Hamiltonian is used for the RT simulation.

A striking feature of the TAS spectrum in Figure 2 is the oscillation of spectral intensity with τ. As shown in Figure S5, this feature is not associated with the variation of the contributing orbitals underlying a particular frequency peak with a time delay. Therefore, nonequilibrium response theory was applied to understand qualitatively the origin of these modulations. Considering that the system consists of only light elements (C, H), a reasonable approach is to use only the nonrelativistic component associated with the real part of the response function in eq 9. As shown in Figure 2d, the DWTA of peak **A** reveals that only two transitions [(15, 16) → (19, 20)] and [(13, 14) → (46, 45)] with orbital energy differences of 0.3305 and 0.6039 au contribute. Therefore, only these transitions appear in the summation over *k*,*j* in eq 9. By discretizing the summation over *m* such that ω_*jm*_ is within the frequency range of [0.0, 0.6 au] and further assuming that the two transitions (I) are of equal probability, i.e., , (II) have a zero phase-factor difference (ϕ_*j*_ – ϕ_*k*_ = 0), and (III) have the same electric dipole transition moments for all *m* (*P*_*v*,*km*_ = 1), we arrive at a simplified formula for the response function, plotted in Figure 2e,
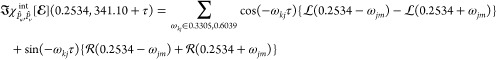
26Even with such simplifications, we were able to mimic the appearance of the intensity oscillation with τ, unique to TAS. In particular, we are capable of capturing the decrease in intensity at the maxima at ω = 0.2534 au, marked by the white dashed line in Figure 2e. Providing more information about the initial phase difference and dipole contributions will enable one to more closely reproduce the spectral feature from response theory.

Next, we focus on the energy region of the core L_2,3_ edge of sulfur in thiophene. The experimental^98^ and simulated ground-state XASs at the nonrelativistic (1c), amfX2C (2c), and Dirac–Coulomb (4c) Hamiltonian levels are presented in Figure 3, clearly demonstrating the importance of including SO coupling effects to reproduce the doublet structure of the spectra (**B**_III_^′^, **C**_III_^′^ versus **B**_II_^′^, **C**_II_^′^). The DWTA analysis of individual peaks (shown in Figures S6 and S7) reveals that the lower-energy feature corresponds to promotion from the S 2p_3/2_, whereas the higher-energy ones are from S 2p_1/2_. Each of the SO split bands is further split due to the molecular field (MF), corresponding to 9e_1/2_, 8e_1/2_ (L_3_ component), and 7e_1/2_ (L_2_ component) molecular orbitals and leading to the lower-energy separated doublets. The MF doublet (**B**′ and **C**′) also appears at the 1c level. The SO split counterpart **A**_II_^′^ of band **A**_III_^′^ is hidden under the more intense **B**_III_^′^ and **C**_III_^′^ doublet. The experimental SO splitting of 1.2 eV is well reproduced by our relativistic Hamiltonian. We would like to draw the attention of the readers to the remarkable agreement between the XAS obtained with our amfX2C and gold-standard Dirac–Coulomb Hamiltonian, with the former being generated at less than one-seventh of the computational cost of the latter. In addition, all other computational characteristics of RT simulations such as the number of microiterations and convergence pattern remain identical for the 2c and 4c regime. Finally, we compute the TAS spectra using a setup described in Table 1 and shown in Figure 4. The importance of incorporating relativistic effects is further reinforced here as evident from the SO split doublet spectral peaks generated using the relativistic Hamiltonian, as seen by comparing Figure 4b and 4c. Again, the resemblance of the TAS simulated at the 2c and 4c levels at τ = 0.0 (in Figure 4a) further gives us confidence to use the modern amfX2C Hamiltonian for larger systems in future studies.

**Figure 3:**
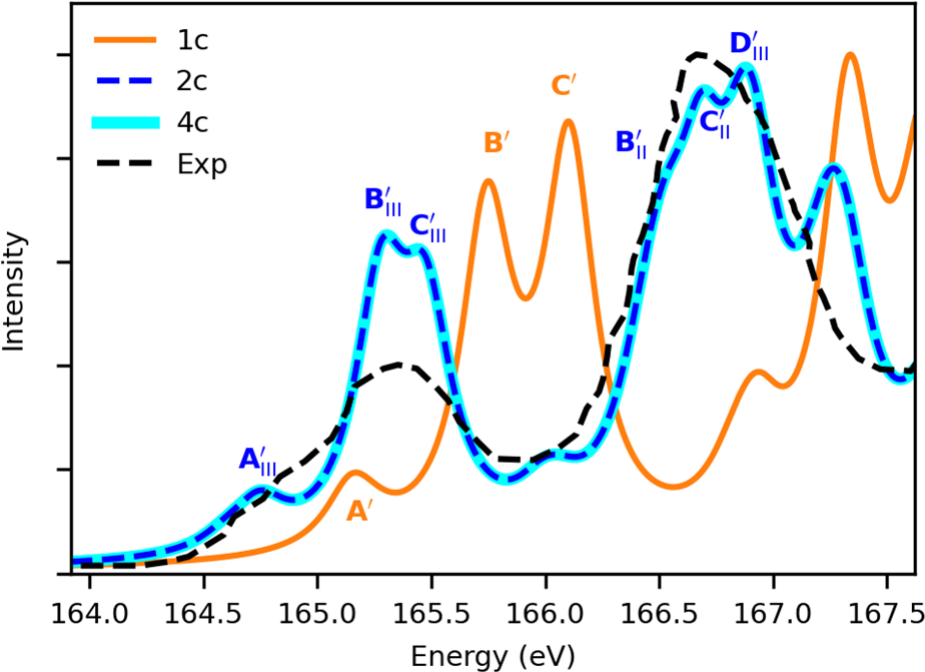
Thiophene. Ground-state L_2,3_-edge X-ray absorption spectra obtained with 1c nonrelativistic (orange), 2c amfX2C (blue), and 4c Dirac–Coulomb (cyan) Hamiltonians using a damping factor of Γ = 0.005 au. The simulated spectra are shifted by +1.42 eV to match the first experimental peak. Experimental results are digitized from ref (98). DWTA analysis of the spectral peaks obtained with 1c and 2c Hamiltonians are shown in Figures S6 and S7, respectively.

**Figure 4:**
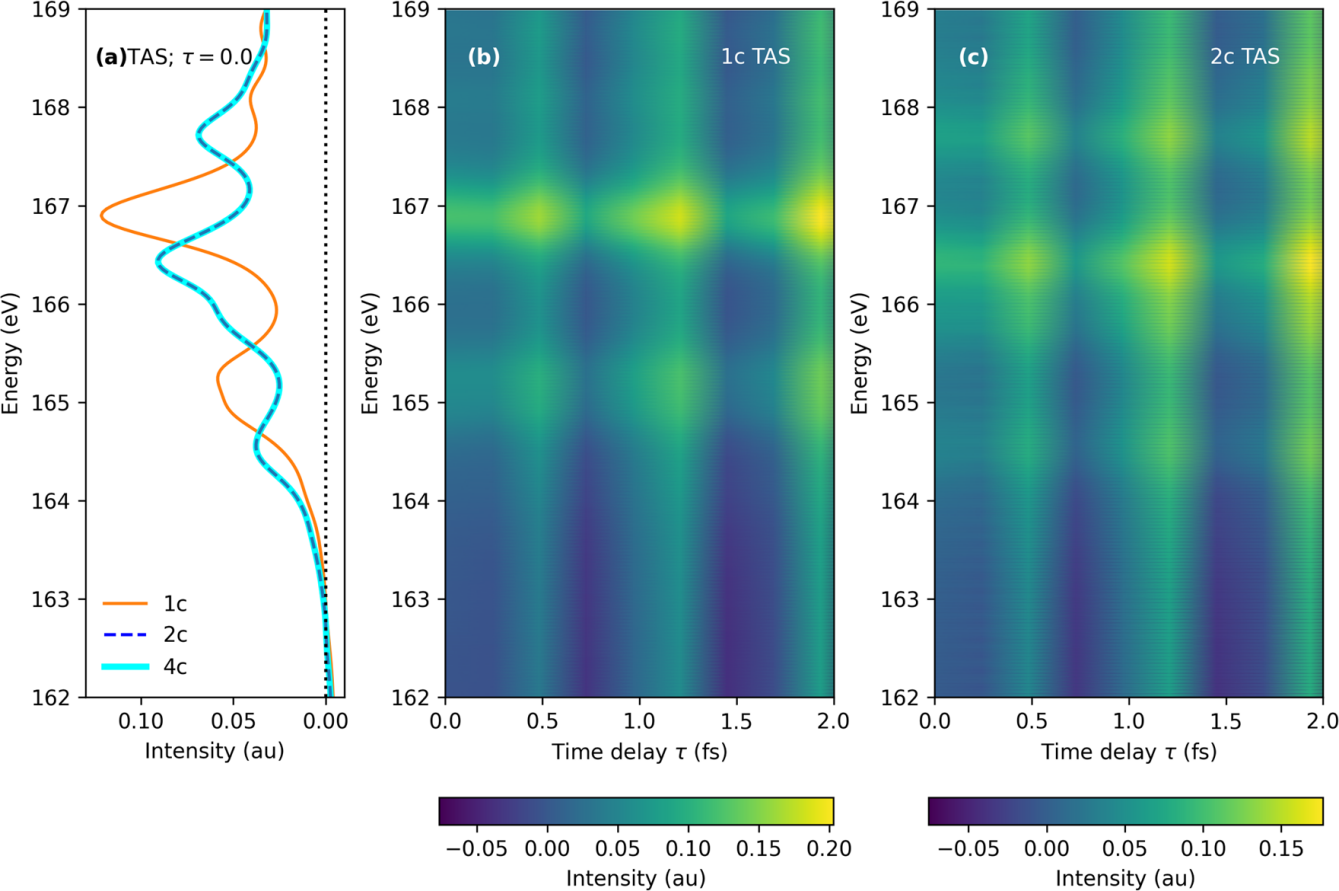
Thiophene. (a) TAS at τ = 0 with 1c nonrelativistic (orange), 2c amfX2C (blue), and 4c Dirac–Coulomb (cyan) Hamiltonians. (b) Variation in TAS spectra with τ obtained with the 1c nonrelativistic Hamiltonian. (c) Variation in TAS spectra with τ obtained with the 2c amfX2C Hamiltonian. All spectra are obtained with a damping factor of Γ = 0.01 au.

In summary, we have formulated and implemented a theoretical approach for the first-principles simulation of TAS spectra based on relativistic RT-TDDFT frameworks, consistently applicable across the periodic table and element-specific core energy regions. Alongside gold-standard four-component methodology, remarkable accuracy and significant computational acceleration were achieved by introducing the amfX2C Hamiltonian within the RT-TDDFT framework. With this, we identify and interpret the unique features associated with TAS spectra, notably the appearance of negative-intensity peaks and oscillations in intensity of a particular energy feature with a pump–probe time delay. These observations were further supported by nonequilibrium response theory, the relativistic generalization of which was also formulated in this letter. Finally, we showcase fingerprints of spin–orbit effects on the X(T)AS spectra near the sulfur L_2,3_ edge of thiophene. We believe that this work constitutes a significant methodological advancement for studying and interpreting transient absorption spectra, applicable to X-ray regimes and/or heavy systems. Work in this direction is currently ongoing in our laboratory.

## Supporting Information Available

Real–time propagator; computational setup; dependence of TAS on the pump pulse carrier frequency; DWTA; and molecular geometries (PDF)
