# Supplementary material for: Accurate Relativistic Real-Time Time-Dependent Density Functional Theory for Valence and Core Attosecond Transient Absorption Spectroscopy
Source: J Phys Chem Lett. 2023 Feb 9;14(7):1714–24. doi: 10.1021/acs.jpclett.2c03599 (PMC9940299; doi:10.1021/acs.jpclett.2c03599)
Supplement: Supplementary file 1 — jz2c03599_si_001.pdf [file jz2c03599_si_001.pdf]

Accurate Relativistic Real-Time Time-Dependent Density Functional Theory for Valence and Core Attosecond Transient Absorption Spectroscopy Supporting information

Torsha Moitra,[†] Lukas Konecny,[†,‡] Marius Kadek,[†,¶,§] Angel Rubio,[‡,‖,⊥] and Michal Repisky[*,†,#]

†*Hylleraas Centre for Quantum Molecular Sciences, Department of Chemistry, UiT The Arctic University of Norway, 9037 Tromsø, Norway*

‡*Max Planck Institute for the Structure and Dynamics of Matter, Center for Free Electron Laser Science, Luruper Chaussee 149, 22761 Hamburg, Germany*

¶*Department of Physics, Northeastern University, Boston, Massachusetts 02115, USA*

§*Algorithmiq Ltd., Kanavakatu 3C, FI-00160 Helsinki, Finland*

‖*Center for Computational Quantum Physics (CCQ), The Flatiron Institute, 162 Fifth Avenue, New York, New York 10010, USA*

⊥*Nano-Bio Spectroscopy Group, Departamento de Física de Materiales, Universidad del País Vasco, 20018 San Sebastian, Spain*

#*Department of Physical and Theoretical Chemistry, Faculty of Natural Sciences, Comenius University, 81499 Bratislava, Slovakia*

E-mail: michal.repisky@uit.no

Contents

S1 Real-time propagator **3**

S2 Computational setup **4**

S3 Dependance of TAS on pump pulse carrier frequency **6**

S4 DWTA **7**

Ethylene: UV-vis 7

Ethylene: TAS 7

Thiophene: XAS 8

S5 Molecular geometries **10**

S1 Real-time propagator

The evolution of a molecular system under the influence of external time–dependent electromagnetic fields is described by the Liouville–von Neumann equation,

$$i\frac{\partial \mathbf{D}(t)}{\partial t} = [\mathbf{F}(t), \mathbf{D}(t)]. \tag{S1}$$

A general solution of the Liouville–von Neumann equation, can be written via an evolution operator $\mathbf{U}(t + \Delta t, t)$ propagating the density matrix from time t to time $t + \Delta t$, as

$$\mathbf{D}(t + \Delta t) = \mathbf{U}(t + \Delta t, t)\mathbf{D}(t)\mathbf{U}^{\dagger}(t + \Delta t, t). \tag{S2}$$

The fact that a *local* propagation between times separated by a short interval Δt has to be considered stems from the fact that Eq. (S1) is non-linear with the Fock $\mathbf{F}(t)$ operator depending on the solution $\mathbf{D}(t)$. However, even for short-time propagation, this non-linearity has to be taken into account when constructing a propagator for the equation. In the present work we approximate the evolution operator by the second-order Magnus propagator

$$\mathbf{U}(t + \Delta t, t) \approx \exp\left[-i\mathbf{F}\left(t + \Delta t/2\right)\Delta t\right] \tag{S3}$$

that preserves time-reversal symmetry, trace, and idempotency of the density matrix even after a truncation. The evolution operator in Eq. (S3) requires a Fock matrix at a future time $t + \frac{\Delta t}{2}$ when a density matrix is not yet available, it has to be constructed by extrapolation from time t and subsequently corrected by interpolation between t and $t + \frac{\Delta t}{2}$. To ensure a correctness and stability of the time evolution, the extrapolation–interpolation step is repeated until self-consistency is reached. The propagator is described in more detail in Repisky et al. [1] where a relativistic real-time TDSCF was introduced into the ReSpect program and the successful propagation algorithm has not been changed since then.

[1]Repisky, Michal, *et al.* J. Chem. Theory Comput. 11(3) (2015): 980-991.

S2 Computational setup

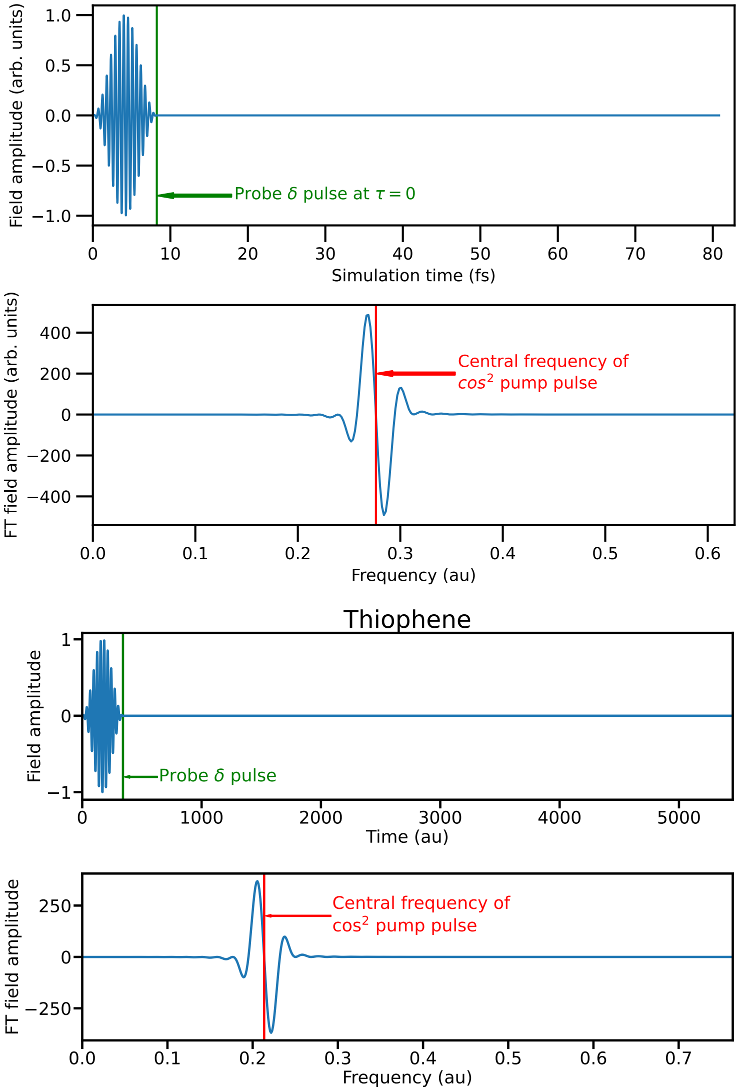

Figure S1: Pulse profile for studying pump-probe TAS.

Table S1: TDDFT excitation energies and oscillator strengths computed using amfX2C Hamiltonian with PBE0 functional and uncontracted aug-cc-pVXZ(C_2H_4: T(C), D(H) and C_4H_4S: T(S), D(C,H))

Molecule	Energy (au)	Oscillator strength			Transition
		x	y	z	
C_2H_4	0.2612	0.0000000000	0.0000000000	0.0697148456	HOMO → LUMO
	**0.2761**	0.3473794966	0.0000000000	0.0000000000	HOMO → LUMO+1
	0.3294	0.0000000000	0.0000000000	0.0090504028	HOMO → LUMO+6
	0.3667	0.0545476832	0.0000000000	0.0000000000	HOMO → LUMO+8
	0.3724	0.0000000000	0.0881552880	0.0000000000	HOMO-1 → LUMO+2
	0.3773	0.0259305238	0.0000000000	0.0000000000	HOMO-1 → LUMO+3
C_4H_4S	**0.2135**	0.0000000000	0.0819208391	0.0000000000	HOMO → LUMO
	0.2220	0.0000000000	0.0000000000	0.0932141506	HOMO-1 → LUMO
	0.2302	0.0136355896	0.0000000000	0.0000000000	HOMO → LUMO+2
	0.2523	0.0190865735	0.0000000000	0.0000000000	HOMO → LUMO+4

S3 Dependance of TAS on pump pulse carrier frequency

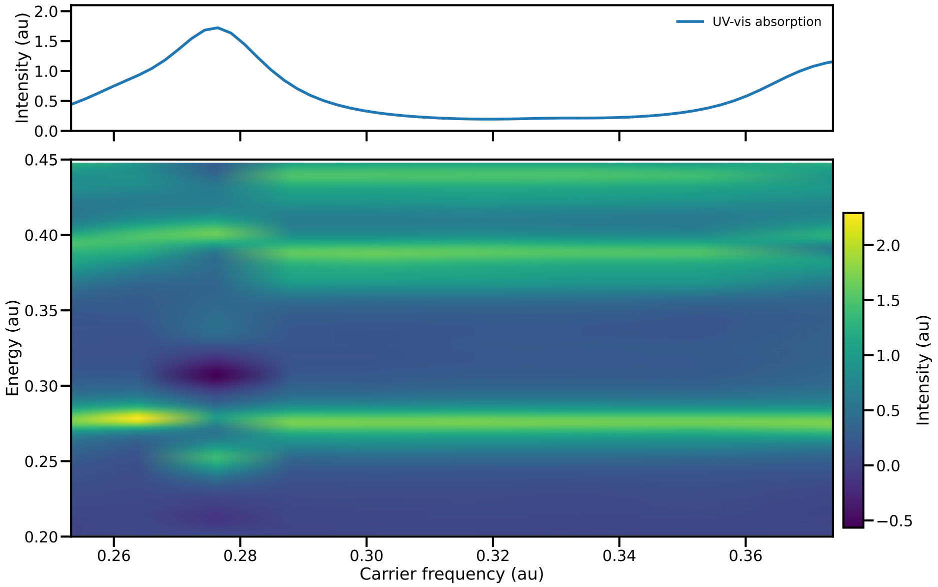

Figure S2: Ethylene– *top:* Uv-vis absorption; *bottom:* TAS with varying pump pulse carrier frequency at $\tau = 0.0$. A 15–cycle $\cos^2$ enveloped pulse is used with an amplitude of 0.01 au. Probe pulse of amplitude 0.01 au is used to investigate the response. The TA spectra is obtained by broadening the spectral lines with a $\Gamma = 0.01$ au

It is evident from above figure that TA spectra is highly sensitive to the carrier frequency of the pump pulse. It governs the excited states populated. An off-resonant carrier frequency does not efficiently depopulate the ground state in order for the TA spectra to differ significantly from the UV-vis absorption spectra. Simultaneously, one can sharpen the time-domain pump pulse, such that the frquency domain signal is broader, leading to effective population of more excited states. However, we do not further dwell into these territory.

S4 DWTA

Ethylene: UV-vis

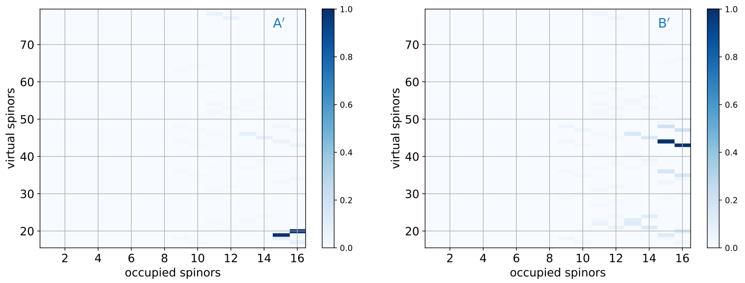

Figure S3: Ethylene – The dipole-weighted transition matrix analysis of UV–visible absorption spectral peaks. A damping parameter $\gamma = 0.01$ au is used. The intensity of the blue color corresponds to the intensity of the particular excitation. The peaks labeled in the legend corresponds to the ones marked in Fig. 2.

Ethylene: TAS

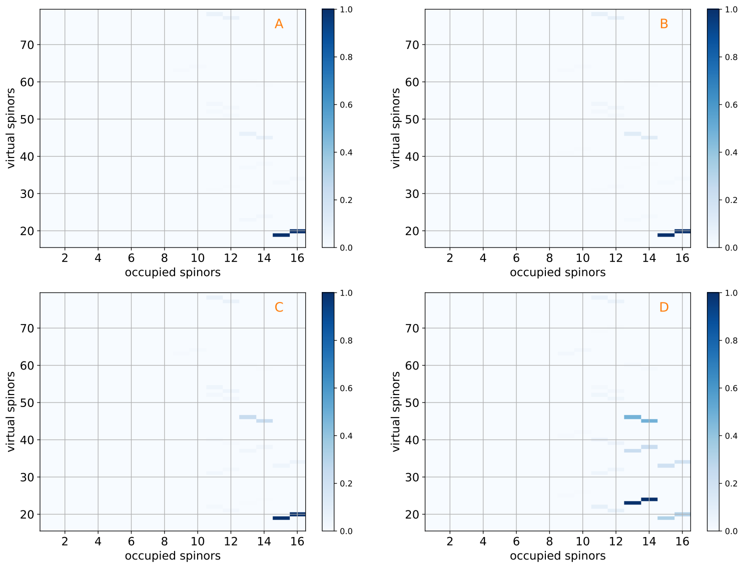

Figure S4: Ethylene – The dipole-weighted transition matrix analysis of transient absorption spectral peaks at $\tau = 0.0$. A damping parameter $\gamma = 0.01$ au is used. The intensity of the blue color corresponds to the intensity of the particular excitation. The peaks labeled in the legend corresponds to the ones marked in Fig. 2.

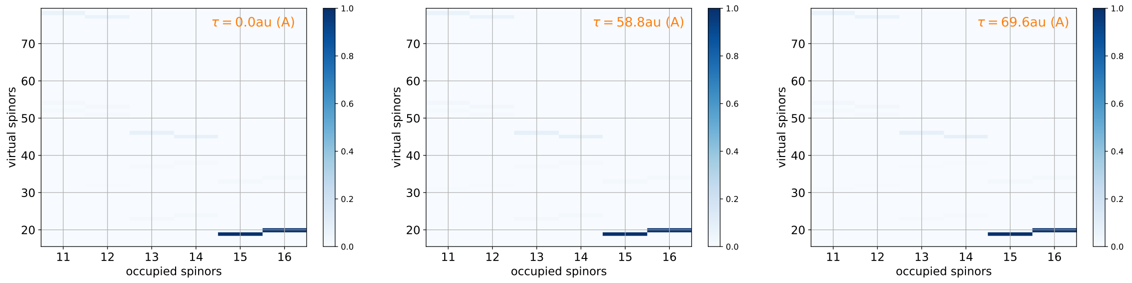

Figure S5: Ethylene: Analysis of peak A in TAS at various τ. The intensity of the blue color corresponds to the intensity of the particular excitation. The peaks labeled in the legend corresponds to the ones marked in Fig. 2.

Thiophene: XAS

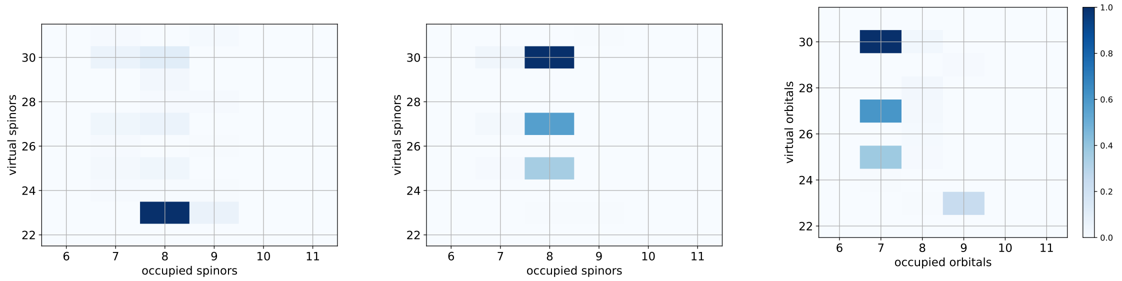

(a) A′ (b) B′ (c) C′

Figure S6: Thiophene: analysis of XAS peaks obtained using non-relativsitic Hamiltonian (1c). The intensity of the blue color corresponds to the intensity of the particular excitation. The peaks are labelled according to Fig. 3.

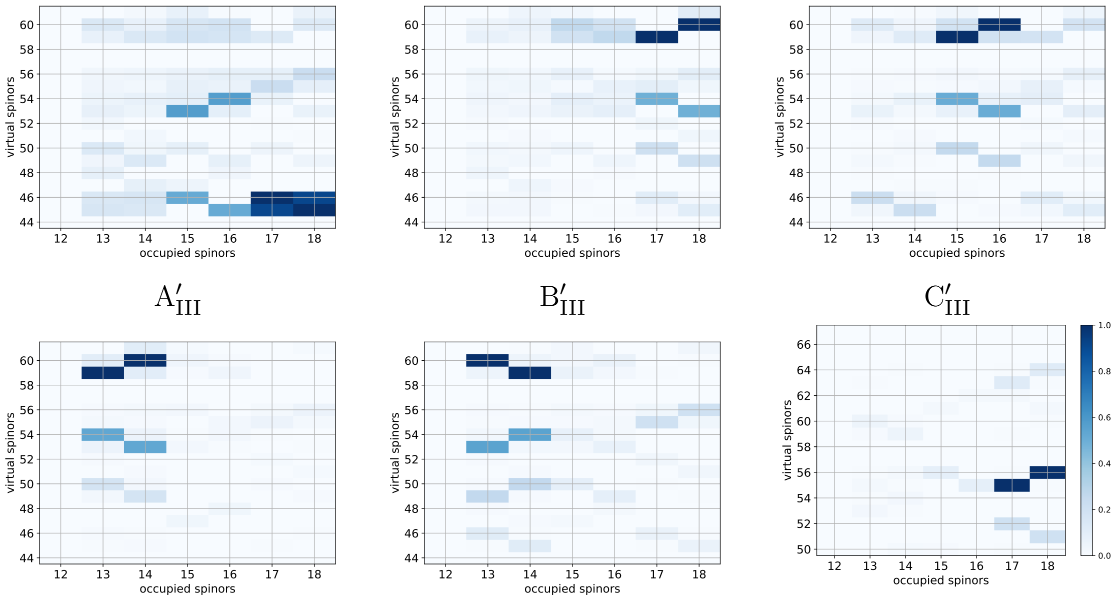

A'_{III} B'_{III} C'_{III}

(a) B'_{II} (b) C'_{II} (c) D'_{III}

Figure S7: Thiophene: analysis of XAS peaks obtained using amfX2C Hamiltonian (2c). The intensity of the blue color corresponds to the intensity of the particular excitation. The peaks are labelled according to Fig. 3.The excitations are always Kramers-paired and form 2×2 sub-matrices. Due to an ambiguity in the phase of these spin–orbitals the internal structure of the 2×2 sub-matrices does not have a physical interpretation.

S5 Molecular geometries

Ethylene (in bohr)

```
C     1.26517  0.000   0.000
C    -1.26517  0.000   0.000
H     2.33230  1.75518 0.000
H    -2.33230  1.75518 0.000
H     2.33230 -1.75518 0.000
H    -2.33230 -1.75518 0.000
```

Thiophene (in Å)

```
S     0.000000   0.000000 -1.158800
C     0.000000   1.228696  0.023308
C     0.000000  -1.228696  0.023308
C     0.000000  -0.704071  1.291013
C     0.000000   0.704071  1.291013
H     0.000000   2.259985 -0.267220
H     0.000000   1.311531  2.176764
H     0.000000  -2.259985 -0.267220
H     0.000000  -1.311531  2.176764
```
